# Worldwide trends in volume and quality of published protocols of randomized controlled trials

**DOI:** 10.1371/journal.pone.0173042

**Published:** 2017-03-15

**Authors:** Belle V. van Rosmalen, Ingo Alldinger, Kasia P. Cieslak, Roos Wennink, Mike Clarke, Usama Ahmed Ali, Marc G. H. Besselink

**Affiliations:** 1 Department of Surgery, Academic Medical Centre, Amsterdam, The Netherlands; 2 Department of General, Visceral and Transplantation Surgery, University of Heidelberg, Heidelberg, Germany; 3 Department of Surgery, University Medical Centre Utrecht, Utrecht, The Netherlands; 4 Northern Ireland Network for Trials Methodology Research, Queen’s University Belfast, Belfast, Northern Ireland; Universitatsspital Basel, SWITZERLAND

## Abstract

**Introduction:**

Publishing protocols of randomized controlled trials (RCT) facilitates a more detailed description of study rational, design, and related ethical and safety issues, which should promote transparency. Little is known about how the practice of publishing protocols developed over time. Therefore, this study describes the worldwide trends in volume and methodological quality of published RCT protocols.

**Methods:**

A systematic search was performed in PubMed and EMBASE, identifying RCT protocols published over a decade from 1 September 2001. Data were extracted on quality characteristics of RCT protocols. The primary outcome, methodological quality, was assessed by individual methodological characteristics (adequate generation of allocation, concealment of allocation and intention-to-treat analysis). A comparison was made by publication period (First, September 2001- December 2004; Second, January 2005-May 2008; Third, June 2008-September 2011), geographical region and medical specialty.

**Results:**

The number of published RCT protocols increased from 69 in the first, to 390 in the third period (*p*<0.0001). Internal medicine and paediatrics were the most common specialty topics. Whereas most published RCT protocols in the first period originated from North America (n = 30, 44%), in the second and third period this was Europe (respectively, n = 65, 47% and n = 190, 48%, *p* = 0.02). Quality of RCT protocols was higher in Europe and Australasia, compared to North America (OR = 0.63, CI = 0.40–0.99, *p* = 0.04). Adequate generation of allocation improved with time (44%, 58%, 67%, *p* = 0.001), as did concealment of allocation (38%, 53%, 55%, *p* = 0.03). Surgical protocols had the highest quality among the three specialty topics used in this study (OR = 1.94, CI = 1.09–3.45, *p* = 0.02).

**Conclusion:**

Publishing RCT protocols has become popular, with a five-fold increase in the past decade. The quality of published RCT protocols also improved, although variation between geographical regions and across medical specialties was seen. This emphasizes the importance of international standards of comprehensive training in RCT methodology.

## Introduction

In 2004, the International Committee of Medical Journals Editors (ICMJE) announced that randomized controlled trials (RCT) should be registered in a public trials registry before the recruitment of the first participant. This registration is now a condition for publication of the final trial results.[1–3]

Although trial registries have many benefits, some authors have suggested that they do not provide full and transparent information about RCT methodology.[4–6] Furthermore, a systematic review highlighted changes between the information in trial registries and the full RCT publication.[7] Publishing RCT protocols gives authors the opportunity to fully explain the rationale and proposed methods for their trial as well as related ethical and safety issues.[7, 8] Although publishing RCT protocols is not a common practice yet, it would potentially benefit trial users and complement the information in trial registries.[9, 10] Moreover, some experts have suggested that it should be mandatory to publish a protocol in order to minimise publication bias, false sample size reporting, switching of endpoints and increase transparency.

In recent years, several studies have addressed trends in the number and methodological quality of RCTs, [7, 11–13] and some addressed the discrepancies between RCTs and their initially published protocols.[14–16] However, no previous study has analysed trends in the publication of study protocols. Thus, little is known about trends in the practice of publishing trial protocols and their methodological quality. Since clinical medicine depends heavily on RCTs, transparency and high quality of RCT protocols is crucial. Therefore, the aim of this study was to assess worldwide trends in the volume and methodological quality of published protocols of RCTs through the first decade of the 21^st^ century.

## Methods

### Aims

This study aimed to analyse trends in the publication of RCT protocols by assessing their volume and methodological quality across specialties and geographic regions.

### Search strategy and selection process

PubMed and EMBASE were searched for trial protocols published in a ten-year period (1 September 2001 to 1 September 2011). In order to interpret the current status of methodological protocol quality, the time interval of ten years was chosen to minimize sampling error, and to ensure sustainability of the results. Also it provides an interesting insight in the development of methodological quality over time. The search syntax was as follows: (design rationale trial AND (randomised OR randomized)) OR (protocol trial AND (randomised or randomized)). All retrieved abstracts were screened according to the inclusion and exclusion criteria by two reviewers (KC, IA). If the relevance was uncertain, the full text of the article was obtained and reviewed. All disagreements were resolved through discussion and reaching consensus by including a third reviewer (MGB).[17] Protocols were included if they described a RCTs, defined as any prospective study assessing the effect of health care interventions in humans, randomly allocated to one of at least two study groups. Studies were excluded when (1) trial results were listed rather than protocols, (2) the study was not a RCT, (3) the study was not a study in humans, (4) the publication was not written in the English language, (5) no abstract was present, (6) no full text was present, and (7) the protocol was published after the study had been completed.

### Study outcomes

The primary study outcome was methodological quality, with as secondary outcome the volume of published protocols. Methodological quality was assessed on two parameters:

1. Individual methodological characteristics: All protocols were appraised according to a list adapted from the Cochrane risk of bias assessment tool and Chan and Altman’s review including the following characteristics [11, 18]:
  - Specification of primary outcome: adequate if primary outcome was explicitly specified in the protocol.
  - Sample size calculation: adequate if performed and reported.
  - Generation of allocation sequence: adequate if method of generation was reported and considered adequate (i.e. computer, random table, shuffle of cards).
  - Concealment of treatment allocation: adequate if method of concealment was reported and considered adequate (i.e. envelopes, central unit for randomization, pharmacy, and independent statistician).
  - Any blinding: adequate if any type of blinding was performed.
  - Double blinding: adequate if both patient and one of the following were blinded: physician, observer, adjudication / consensus committee.
  - Type of analysis: adequate if intention-to-treat analysis was explicitly mentioned.
2. High vs. low quality designs: a trial was designated as ‘high quality’ if all three of the following methodological items were adequately reported: generation of allocation, concealment of allocation and intention-to-treat analysis. Blinding was not included as an item. Some have claimed that the role of blinding is overstated [19, 20]. Blinding may be impossible in some surgical trials.[21–24] Estimating correct implementation (or legitimate non-implementation) of blinding will not be possible, considering the great variety of possibilities to implement blinding among medical specialties. Therefore concealment of sequence generation was chosen instead, since it is a more generalizable parameter.[25]

### Data extraction and definitions

The following geographical, publishing and epidemiological characteristics were extracted: geographical region, specialty (based on the corresponding author and divided into the following (arbitrary) categories: Internal medicine and paediatrics, primary care, surgery (including subspecialties) and other), number of study centres, study arms (two arms, or three and more), number of randomized patients, trial design, funding (any kind of involvement of the industry was stated as commercial), presence of written informed consent, presence of a data safety monitoring board and plan for dealing with adverse events.

### Data analysis

Characteristics and outcomes of included protocols were compared for three approximately equal periods: September 2001 to December 2004, January 2005 to May 2008 and June 2008 to September 2011.

Because of the search strategy used, only study protocols published in the last 4 months of the year 2001 and the first 8 months of the year 2011 were included. A random sample of publications from those years was added to the database as substitute for the missing months in 2001 and 2011. This was conducted according to the following manner: the number of publications per month of included protocols that were scored on quality in 2001 and 2011 respectively was calculated. This mean was multiplied by the number of missing months (8 in 2001 and 4 months in 2011 respectively). This resulted in 3 protocols being added to 2001 and 40 to 2011. The added protocols were randomly selected from period 1 and period 3, respectively.

Subgroup analyses were based on geographical region and medical specialty. The rational for examining geographical variation as well as medical specialties was that previous research demonstrated differences in methodological quality of surgical trials between continents.[26] Dichotomous outcomes were presented as the number (percentage) of events, whereas medians and interquartile ranges were used for continuous data. Study groups were compared by Fisher exact, χ2 and Mann-Whitney U tests, as appropriate. A *p*-value of <0.05 was considered significant. The odds ratio (OR) with the corresponding 95% confidence intervals (95% CIs) was calculated for comparison of methodological quality between subgroups by means of univariate and multivariate logistic regression. All variables were included in the univariate analysis. Variables showing potential association (p<0.2) in the univariate analysis were subsequently included in the multivariate analysis.[27] IBM SPSS Statistics for Windows Version 20.0 (IBM Corp., Armonk, NY, USA) was used for all statistical analysis.

## Results

### Selection process

Our search identified a total of 11 782 records. The selection process is depicted in figure A of the supporting information. The screening of the titles resulted in the selection of 6074 potentially relevant publications, and after screening by title and abstract, 615 publications remained. After final selection of full-text, 553 eligible protocols were identified. A random sample of 43 protocols was added to the database, resulting in a total of 596 protocols.

### General characteristics and volume

Table 1 shows the clinical and epidemiological characteristics of the included protocols.

**Table 1:** Baseline and general characteristics of 596 published protocols of RCTs.

General characteristics	First period Sept 2001-Dec 2004	Second period Jan 2005-May 2008	Third period Jun 2008-Sept 2011	*p*-value
(number) (percentage)	(n) (%)	(n) (%)	(n) (%)	
Total	69 (12)	137 (23)	390 (65)	<0.0001
Region;				
Europe	24 (35)	65 (47)	190 (49)	
North America	30 (44)	46 (34)	103 (26)	
Australasia	11 (16)	25 (18)	86 (22)	
Other	4 (6)	1 (1)	11 (3)	0.02
Specialty;				
Internal medicine and paediatrics	35 (51)	75 (55)	164 (42)	
Primary care	23 (33)	38 (28)	115 (40)	
Surgery	6 (9)	19 (14)	41 (11)	
Other	5 (7)	5 (4)	30 (8)	0.06
Participating countries;				
Single country	42 (61)	94 (69)	312 (80)	
Two or more countries	22 (32)	31 (23)	68 (17)	
Missing	5 (7)	12 (9)	10 (3)	0.006
Participating centres;				
Single	8 (12)	28 (20)	94 (24)	
Multi	61 (88)	109 (80)	296 (76)	0.06
Funding;				
Commercial	33 (48)	46 (34)	113 (29)	
Non-commercial	36 (52)	91 (66)	277 (71)	0.008
Trial design;				
Parallel	56 (81)	120 (88)	325 (83)	
Other/Mixed	13 (19)	17 (12)	65 (17)	0.39
Number of study arms reported;				
Two arms	43 (62)	105 (77)	299 (77)	
Three or more arms	26 (38)	32 (23)	90 (23)	0.032
Number of randomized patients;	640	358	336	
Median (IQR)	(308–2741)	(197–852)	(140–900)	
Written informed consent present;	41 (59)	106 (77)	287 (74)	0.02
Data safety Committee present;	37 (54)	57 (42)	139 (37)	0.02
Plan for adverse events present;	17 (25)	50 (37)	145 (37)	0.13

Legend: This table describes all general characteristics of the included protocols, divided period by of publication. Study groups were compared by Fisher exact, χ2 and Mann-Whitney U tests, as appropriate. A *p*-value of <0.05 was considered significant. IQR = inter quartile range.

The number of published protocols increased substantially over time, with 69, 137 and 390 published in the three periods, respectively (*p*<0.0001) (figure A in the supporting information, and figure B of the supporting information). This constitutes a five-fold increase between the first and third period. In the first period, most published RCT protocols originated from North America, n = 30 (44%), while Europe was the most common in the second and third periods, n = 65 (47%) and n = 190 (48%), respectively.

‘Internal medicine and paediatrics’ was the most common specialty topic for RCTs of the four categories used, in all periods. Overall an increase in the absolute number of protocols was observed, although the numbers remained relatively low in the surgical category (n = 6, 19, 41). The proportion of non-industry-funded trials almost doubled between the first and last time period from 27 (39%) to 254 (65%), (*p*<0.0001). There was a decrease in the reporting of the use of a data safety committee: from 54% in the first period to about 36% in the third period, (*p* = 0.02).

### Methodological quality

Methodological quality of the included protocols is presented in Table 2.

**Table 2:** Quality characteristics of published protocols.

Individual quality characteristics	First period Sept 2001-Dec 2004 n = 69	Second period Jan 2005-May 2008 n = 137	Third period Jun 2008-Sept 2011 n = 390	*p*-value
(number) (percentage)	(n) (%)	(n) (%)	(n) (%)	
Primary outcome specified;	65 (94)	134 (98)	380 (97)	0.3
Sample size calculation reported;	64 (93)	123 (90)	359 (92)	0.7
Adequate generation of allocation;	30 (44)	79 (56)	260 (67)	0.001
Concealment of allocation;	26 (38)	72 (53)	215 (55)	0.03
Any Blinding;	50 (73)	90 (66)	268 (69)	0.60
Blinding—patient;				
Yes	27 (39)	50 (37)	103 (26)	
No	35 (51)	75 (55)	274 (70)	
Unclear	7 (10)	12 (9)	13 (3)	0.02
Blinding—observer;				
Yes	33 (48)	67 (49)	235 (60)	
No	22 (32)	48 (35)	122 (31)	
Unclear	14 (20)	22 (16)	33 (9)	0.02
Blinding—physician;				
Yes	25 (36)	37 (27)	76 (20)	
No	36 (52)	86 (63)	297 (76)	
Unclear	8 (12)	14 (10)	17 (4)	0.005
Blinding—adjudication committee;				
Yes	17 (25)	22 (16)	40 (10)	
No	52 (75)	114 (83)	350 (90)	
Unclear	0 (0)	1 (1)	0 (0)	0.003
Double blinding;	27 (39)	49 (36)	97 (25)	0.008
Prospective subgroup analyses;	16 (23)	37 (27)	80 (21)	0.3
Intention to treat analyses;	43 (62)	97 (71)	293 (75)	0.08
High quality protocols; *	18 (26)	43 (31)	143 (37)	0.2

Legend: This table describes all characteristics concerning methodological quality, subdivided by period of publication. Study groups were compared by Fisher exact, χ2 and Mann-Whitney U tests, as appropriate. A *p*-value of <0.05 was considered significant.

* defined as presence of the following 3 criteria: adequate generation of allocation, concealment of allocation and intention-to-treat analysis.

The proportion of high quality protocols increased non-significantly across the study periods: 18 (26%), 43 (31%) and 143 (37%), (*p* = 0.17). Adequate methods for generation and concealment of allocation improved significantly over time (*p* = 0.03). Blinding was applied relatively frequent (about 70%) throughout the study periods, while the use of a blinded observer increased from 48% in the first period to 67 (60%) in the third (*p* = 0.02). The number of studies attempting to blind patients decreased significantly over time: from 39% and 37% in the first two periods, respectively, to 26% in the third (*p* = 0.02). The rate of double-blinding also decreased accordingly, from 39% and 36% to 25%, respectively (*p*<0.008). There was a non-significant increase in explicit intention-to-treat analysis, from 62% in the first period to 75% in the third (*p* = 0.08).

### Subgroup and regression analysis

Subgroup analysis by geographic region is presented in Table 3.

**Table 3:** Subgroup analyses by region and speciality.

Individual quality characteristics	Europe n = 279	North America n = 179	Australasia n = 122	*p*-value
(number) (percentage)	(n) (%)	(n) (%)	(n) (%)	
Sample size calculation reported;	253 (91)	161 (90)	116 (95)	0.3
Primary outcome specified;	274 (98)	167 (93)	122 (100)	0.001
Generation of allocation;	183 (66)	94 (53)	79 (65)	0.01
Concealment of allocation;	154 (55)	77 (43)	72 (59)	0.009
Some blinding;	182 (65)	125 (70)	88 (72)	0.3
Blinding–patient;	71 (25)	66 (37)	34 (28)	0.03
Blinding–observer;	165 (59)	91 (51)	72 (59)	0.2
Blinding–physician;	47 (17)	61 (34)	22 (18)	<0.0001
Blinding–adjudication committee;	20 (7)	37 (21)	17 (14)	<0.0001
Double blinding;	68 (24)	65 (36)	31 (25)	0.02
Prospective subgroup analyses;	68 (24)	40 (22)	23 (19)	0.5
Intention to treat analyses;	221 (79)	108 (60)	93 (76)	<0.0001
High quality protocols; *	99 (36)	45 (25)	53 (43)	0.003
Individual quality characteristics	Internal & paediatrics n = 274	Primary care n = 216	Surgery n = 66	*p*-value
(number) (percentage)	(n) (%)	(n) (%)	(n) (%)	
Sample size calculation reported;	256 (93)	194 (90)	61 (92)	0.34
Primary outcome specified;	271 (99)	206 (95)	64 (97)	0.06
Generation of allocation;	153 (56)	140 (65)	48 (73)	0.02
Concealment of allocation;	120 (44)	127 (59)	38 (58)	0.002
Some blinding;	213 (78)	129 (60)	33 (50)	<0.0001
Blinding–patient;	107 (39)	35 (16)	18 (27)	<0.0001
Blinding–observer;	156 (57)	120 (56)	29 (44)	0.16
Blinding–physician;	89 (33)	24 (11)	13 (20)	<0.0001
Blinding–adjudication committee;	66 (24)	7 (3)	3 (5)	<0.0001
Double blinding;	104 (38)	33 (15)	18 (27)	<0.0001
Prospective subgroup analyses;	68 (25)	41 (19)	14 (21)	0.30
Intention to treat analyses;	194 (71)	158 (73)	49 (74)	0.78
High quality protocols; *	77 (28)	80 (37)	29 (44)	0.02

Legend: This table describes the subgroup analysis for methodological quality characteristics subdivided by specialty or region. Study groups were compared by Fisher exact, χ2 and Mann-Whitney U tests, as appropriate. A *p*-value of <0.05 was considered significant.

* Presence of the following three criteria: adequate generation of allocation, concealment of allocation and intention-to-treat analysis.

Adequate generation and concealment of allocation were equally frequent in RCT protocols from Europe and Australasia (around 60%), while less often so in protocols from North America (52% and 43%, respectively, *p*≤0.01). A similar trend was observed for adequate type of planned analyses (i.e. explicitly intention to treat): 79% and 76% for European and Australasian, respectively, compared to 60% for North American protocols (*p*<0.0001). However, for blinding, North America achieved the highest percentages on practically all parameters. This is reflected in a double-blinding proportion of 36% compared to 24% and 25% for European and Australasian protocols, respectively (*p* = 0.02).

Subgroup analyses comparing the three most common specialties (Internal medicine and paediatrics, primary care and surgery) were performed (Table 3). A significant difference in adequate generation of allocation and adequate concealment allocation was observed with the highest percentage achieved by surgery protocols (*p* = 0.02 and *p* = 0.002, respectively). On the other hand internal medicine and paediatrics consistently scored the highest percentage for blinding. The highest percentage of high quality protocols was in surgery (44%), (*p* = 0.018).

Univariate regression analysis shows origin from North America to be negatively associated with methodological quality, while origin from Europe, primary care or surgery as specialty, presence of informed consent and presence of a plan for adverse event were predictors for high methodological quality. In multi-variate analysis, all these factors were confirmed as independent predictors for methodological quality (Table 4).

**Table 4:** Regression analyses for high protocol quality.

Univariate regression analyses; Characteristics	(n)	Odds for high quality. OR (95% CI)	*p*-value
Region;			
Europe	279	1	-
North America	179	0.61 (0.40–0.93)	0.02
Australasia	122	1.40 (0.91–2.20)	0.13
Specialty;			
Internal medicine and Paediatrics	274	1	-
Primary care	216	1.51 (1.03–2.20)	0.04
Surgery	66	2.01 (1.15–3.47)	0.01
Number of participating countries; Single country	448	0.86 (0.57–1.31)	0.5
Participating centres; Single	130	0.68 (0.44–1.04)	0.08
Funding; Commercial	192	1.14 (0.79–1.64)	0.5
Trial design; parallel	501	1.15 (0.72–1.85)	0.6
Two study arms reported; Yes	447	0.95 (0.64–1.41)	0.8
Written informed consent; Yes	434	1.50 (1.01–2.23)	0.04
Data safety committee present; Yes	233	1.18 (0.83–1.66)	0.4
Plan adverse events; Yes	212	1.69 (1.19–2.40)	0.003
Multivariate regression analyses; Characteristics	(n)	Odds for high quality. OR (95% CI)	*p*-value
Region;*			
Europe	257	1	
North America	168	0.63 (0.40–0.99)	0.04
Australasia	117	1.30 (0.82–2.06)	0.3
Specialty;			
Internal and paediatrics	262	1	
Primary care	214	1.57 (1.04–2.36)	0.03
Surgical specialties	66	1.94 (1.09–3.45)	0.02
Written informed consent; Yes	434	1.42 (0.90–2.23)	0.13
Plan adverse events; Yes	212	1.81 (1.22–2.68)	0.003

Legend: This table describes odds ratio (OR) with the corresponding 95% confidence intervals (95% CIs), and was calculated for comparison of methodological quality between subgroups by means of univariate and multivariate logistic regression. A *p*-value of <0.05 was considered significant.

*The small number of protocols from regions labelled as “other” (e.g. Africa and South America) were not included in the multivariate analysis, but are reported in Table 1.

## Discussion

This first systematic empirical literature-based study on volume and quality of RCT protocols found a five-fold increase in the number of RCT protocols published over a ten-year period. Although the overall quality of published protocols improved, there were differences between continents, with protocols from Australasia and Europe being of higher quality than those from North America. This empirical literature-based study also found medical specialty to be correlated with the quality of published RCT protocols. Both primary care and surgical trials were associated with significantly higher quality compared to internal medicine and paediatric protocols. However, the confidence intervals of these parameters were relatively broad. Therefore it cannot be excluded that confounders that were not accounted for in the multivariate analysis contributed to the overall significance.

Previous studies have identified similar trends for the volume of published RCTs, as for published protocols.[26, 28–32] Whether protocol publication is increasing in popularity, or whether the augmentation in volume can be subscribed as a direct consequence of the increased amount of RCTs remains uncertain. In contrast with the current study, previous studies found published RCTs from Australasia to have the lowest rates of adequate reporting.[26, 33–36] The higher quality of surgical trials is remarkable especially since surgery used to have a reputation of being based on tradition rather than scientific research.[37, 38] A possible explanation for this phenomenon could be that due to the increasing rate of technological innovation more (e.g. minimally invasive) techniques have become available which allow for randomized comparisons. Moreover, increased awareness of the importance of surgical trials and enhanced training in trial methodology may have attributed to this improvement, but data are lacking. The recent IDEAL (Idea, Development, Exploration, Assessment, Long-term Follow-up) framework for surgical innovation may provide guidance for further improvement of trials on surgical interventions.[39, 40]

A troubling, and yet unexplained, finding is the apparent decrease in the use of a data safety committee from 54% in the first period to 36% in the third period. Close monitoring of this development is imperative.[41] In fact, the presence of a plan to handle adverse events seemed to be the strongest indicator for high quality RCT protocols. This might be explained by the importance of having such a plan is especially important in trials with a high degree of trial complexity; such trials will have been designed more carefully.[42] The intention of gaining written informed consent was also found to be a marker for high protocol quality. It seems that evidence-based guidance on how to design and perform RCTs would be welcomed. The Trial Forge platform and the SPIRIT guidelines (Standard Protocol Items: Recommendations for Interventional Trials) could be instrumental in this aspect as it strives to provide a systematic approach to improving trials and their protocols.[43, 44]

A shortcoming of our study is that the quality of the protocol does not automatically translate into the quality of the RCT. Although previous studies have compared the quality of RCTs with the quality of their protocols, such studies are scarce. Furthermore, they have used small samples and some of their results are contradictory.[9, 15, 45] Whether the trials described in the protocols in our study will be performed and published as designed, should be investigated further. This might reveal important insights in the life cycle of RCTs, and would allow prospective evaluation of factors that might be related to early termination of RCTs and non-publication. Another shortcoming of this study is that instead of the SPIRIT guidelines, the Cochrane risk of bias tool was used. The use of the SPIRIT item check list would have expanded the analysis. The drawback of the SPIRIT checklist, however, is that it covers over 50 items, including recommendations on version identifiers and statements regarding who obtained informed consent and who have access to the final data. These data are often not available in the published protocols. Therefore, a more selective approach was chosen in which a selected list of items was evaluated with empirical evidence showing their importance in that they affect final outcomes of RCTs. Additionally, the fact that these items have been used previously in several studies allows comparison between studies.

Medical specialties were subdivided into three fairly broad and subjective groups, in order to compare and contrast our findings across this range of subspecialties. This might have resulted in a loss of detail.[28, 29] Furthermore, only protocols published in English were included, which may have led to an underestimation of the number of published protocols, assuming that some are published in other languages. The inclusion of protocols was not limited to the top listed medical journals, which is a strength of this study. Also our review covers all medical specialties, which makes our study results generalizable.

In conclusion, this systematic review found a five-fold increase in the number of published study protocols in the past decade. The methodological quality of the protocols improved during the same period but varies greatly between regions and medical specialties, which suggests that different regions and medical specialties may face different challenges when seeking to improve the quality of RCTs. Nevertheless, it is important to strive for such improvements, given the importance of RCTs and systematic reviews of them as a source of reliable and robust evidence on the effects of healthcare interventions. Comprehensive training in RCT methodology, as for example is already offered in a master programme at the University of Oxford, amongst others, could benefit responsible conduct and reporting of RCTs greatly. The involvement of international medical societies in developing standards for training could enhance RCT quality improvement world-wide.

## Supporting information

S1 File **Figure A in:** Flow chart of systematic search strategy in PubMed and EMBASE. **Figure B in S1 File:** Volume of published protocols in number, per region; This figure describes the number of published protocols (listed on the x-axis) for different regions, sub divided in three time periods (listed on the y-axis).
(DOCX)

S2 File **Figure C in S2 File:** Filled in Prisma checklist.
(DOC)
