# Correction: Worldwide trends in volume and quality of published protocols of randomized controlled trials

**DOI:** 10.1371/journal.pone.0187389

**Published:** 2017-10-26

**Authors:** Belle V. van Rosmalen, Ingo Alldinger, Kasia P. Cieslak, Roos Wennink, Mike Clarke, Usama Ahmed Ali, Marc G. H. Besselink

The sixth author’s name is incorrectly abbreviated in the citation. The correct citation is: van Rosmalen BV, Alldinger I, Cieslak KP, Wennink R, Clarke M, Ahmed Ali U, et al. (2017) Worldwide trends in volume and quality of published protocols of randomized controlled trials. PLoS ONE12(3): e0173042. https://doi.org/10.1371/journal.pone.0173042
