# Supplementary material for: Worldwide trends in volume and quality of published protocols of randomized controlled trials
Source: PLoS One. 2017 Mar 15;12(3):e0173042. doi: 10.1371/journal.pone.0173042 (PMC5351864; doi:10.1371/journal.pone.0173042)
Supplement: S1 File — Figure A in: Flow chart of systematic search strategy in PubMed and EMBASE. Figure B in S1 File: Volume of published protocols in number, per region; This figure describes the number of published protocols (listed on the x-axis) for different regions, sub divided in three time periods (listed on the y-axis). (DOCX) [file pone.0173042.s001.docx]

**SUPLEMENTARY S1**

**Figure A. Flow chart of systematic search strategy in PubMed and EMBASE.**

Random sample
(n=43)

Protocols included in qualitative synthesis
(n = 596)

Eligible protocols
(n =553)

Full-text articles excluded, with reasons
Study results rather than protocols (n =34)
No full text (n =12)
No RCT (n =10)
Economic analysis rather than protocol (n =2)
Multiple trials (n =2)
Outside study period (n =2)

Records screened
(n =6074)

Full-text articles assessed for eligibility
(n =615)

Records identified through database searching
(n =11,782)

Records excluded
(n = 5459)

Records after duplicates removed
(n = 6074)


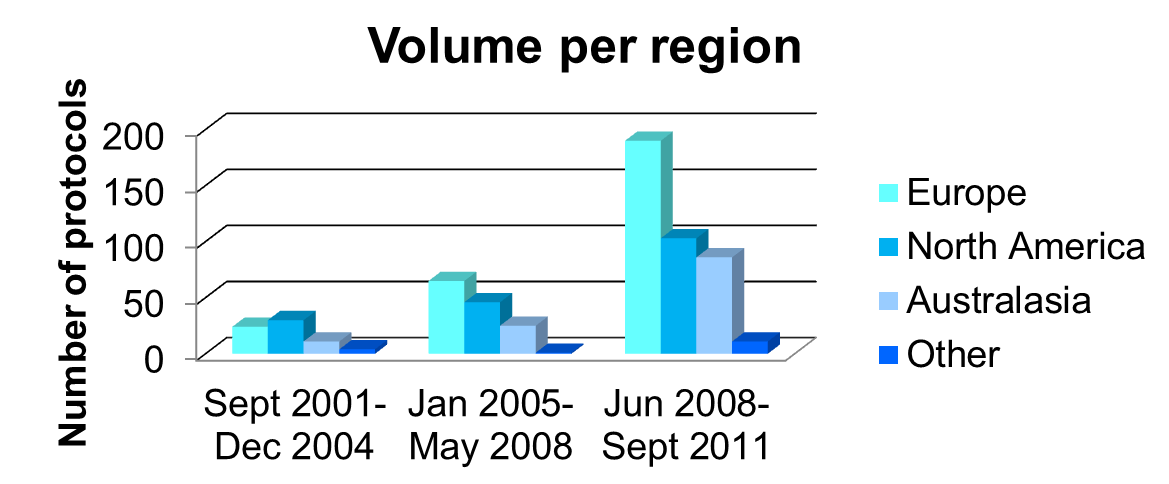
**Figure B. Volume of published protocols in number, per region.**

This figure describes the number of published protocols (listed on the x-axis) for different regions, sub divided in three time periods (listed on the y-axis).
